# Implementation of a structured early oral supportive-care pathway and early oral health recovery after oral cancer resection and free-flap reconstruction: a prospective observational cohort study

**DOI:** 10.3389/fonc.2026.1981326

**Published:** 2026-10-20

**Authors:** Tsai-Mei Huang, Yun Chih Chen, Yu-Che Cheng, Chi-Yuan Yeh, Yu-Kang Chang, Mei-Wen Nian, Tsay-I Chiang, Stella Chin-Shaw Tsai

**Affiliations:** 1 Department of Nursing, Tungs’ Taichung MetroHarbor Hospital, Taichung, Taiwan; 2 Department of Radiation Oncology, Tungs’ Taichung MetroHarbor Hospital, Taichung, Taiwan; 3 Department of Medical Research, Tungs’ Taichung MetroHarbor Hospital, Taichung, Taiwan; 4 Department of Nursing, Jenteh Junior College of Medicine, Nursing and Management, Miaoli, Taiwan; 5 Department of Otolaryngology, Tungs’ Taichung MetroHarbor Hospital, Taichung, Taiwan; 6 Department of Nursing, Hungkuang University, Taichung, Taiwan; 7 Department of Post-Baccalaureate Medicine, National Chung Hsing University, Taichung, Taiwan; 8 College of Life Sciences, National Chung Hsing University, Taichung, Taiwan

**Keywords:** caregiver participation, free-flap reconstruction, implementation, nursing care, oral cancer, oral health recovery, postoperative care, supportive care

## Abstract

**Background:**

Patients undergoing oral cancer resection and free-flap reconstruction often have limited capacity for independent oral hygiene during the immediate postoperative period. Standardized bedside oral supportive-care pathways may help organize early postoperative care, but their implementation and accompanying early oral-health recovery have not been well characterized prospectively.

**Methods:**

This prospective, single-center observational cohort study included 36 adults undergoing oral cancer resection with free-flap reconstruction who received a structured early postoperative oral supportive-care pathway beginning on the day of surgery. The primary outcome domains for the present manuscript were pathway implementation, short-term tolerability, and early oral-health recovery from postoperative day 3 (POD3) to POD13. Clindamycin-based and chlorhexidine-based formulations were both used in routine clinical care and were not assigned by the research team; formulation-specific comparisons were secondary and exploratory.

**Results:**

All 36 participants completed both Oral Assessment Scale for Post-Operational Patients with Oral Cancer (OASPOCa) assessments, and 32 (88.9%) were recorded as completing all scheduled daily oral-care sessions in the archived adherence record; four participants had one missed-session record. No clinically significant formulation-related intolerance or discontinuation was recorded. Mean total OASPOCa score decreased from 14.97 (SD 2.79) on POD3 to 11.44 (SD 1.65) on POD13, corresponding to a mean within-participant improvement of 3.53 points (SD 3.15; 95% CI, 2.46–4.59; P < 0.001). At least some participants improved in each of the 10 OASPOCa domains, while others remained unchanged or worsened. In secondary exploratory analyses, the between-formulation difference in mean improvement was 0.39 points (95% CI, −1.78 to 2.55; P = 0.717).

**Conclusion:**

The structured pathway was implemented with high recorded participant-level adherence and was accompanied by multidimensional early oral-health recovery; no clinically significant formulation-related intolerance or discontinuation was recorded. Because oral care began before the first formal assessment and no untreated or usual-care control group was included, the observed change represents temporal postoperative recovery rather than causal pathway efficacy. No clear overall advantage of either routinely used oral-care formulation was demonstrated.

## Introduction

Major oral cancer resection with free-flap reconstruction creates a demanding early recovery environment in which routine self-care can become difficult or temporarily impossible. Newly reconstructed intraoral tissue, surgical sutures, edema, blood and secretion accumulation, pain, restricted mouth opening (1), airway-related devices, and nasogastric-tube feeding can all interfere with oral cleansing and increase dependence on nurses and family caregivers. At the same time, these procedures are complex, frequently involve a clean-contaminated operative field, and require coordinated postoperative management. For these reasons, oral supportive care is not merely a comfort measure but a potentially important component of a standardized perioperative recovery pathway (2, 3). Postoperative supportive care also occurs within a broader oncologic continuum in which local recurrence remains an important clinical concern after oral cancer surgery (4).

The broader perioperative literature supports the clinical importance of oral care, although the optimal postoperative bedside protocol remains uncertain. A 2024 systematic review and meta-analysis of patients undergoing cancer surgery found that professional oral hygiene care was associated with lower postoperative pneumonia and surgical-site infection rates and shorter hospital stay, while also noting uncertainty regarding the independent contribution of specific nursing oral-care components (5). In a retrospective cohort of 183 patients undergoing head and neck free-flap reconstruction, professional preoperative oral care was independently associated with lower odds of surgical-site infection (odds ratio 0.39) (2). A separate head and neck cancer cohort linked inadequate plaque control with postoperative pneumonia risk (6). A prospective comparative study after oral and maxillofacial free-flap surgery found that adding 3% sodium bicarbonate saline to postoperative oral care reduced oral candidiasis, illustrating that solution-specific postoperative oral-care strategies have been evaluated in this setting (7). Postoperative infection is also clinically consequential: surgical-site infection after oral cavity cancer resection and reconstruction has been associated with substantially greater plate exposure (8), while a prospective multicenter study of oncological microvascular head and neck reconstruction linked healthcare-associated infections with reoperation, prolonged hospitalization, and delayed postoperative radiotherapy (9). Collectively, these studies establish the clinical relevance of perioperative oral hygiene, but much of the evidence emphasizes preoperative professional care or infection-related outcomes rather than the condition of the oral cavity during the first postoperative days.

The immediate postoperative period presents a different clinical problem. Patients may be unable to brush, rinse, expectorate, or inspect the oral cavity independently, while frequent tube feeding and secretion retention can further complicate local care. Enhanced Recovery After Surgery recommendations support multimodal and standardized perioperative pathways for major head and neck cancer surgery, while acknowledging that evidence for individual pathway components is uneven (3). Oral care remains particularly variable. In a multicenter survey of intensive-care nurses caring for postoperative oral cancer patients, assessment standards, operating procedures, oral-care products, and continuing education were inconsistent, and substantial training needs were reported (10). Randomized, quasi-experimental, and systematic evidence on early swallowing and oral-motor interventions indicates that targeted supportive care can improve postoperative swallowing-related and functional outcomes after oral cancer surgery and free-flap reconstruction (11–15). Longer-term prospective rehabilitation and free-flap outcome studies further show that swallowing, speech, and quality of life may remain impaired or require structured rehabilitation beyond the immediate postoperative period (16–20). These observations support a bedside protocol that defines when care begins, how often it is delivered, who performs it, how technique is supervised, and how recovery is assessed across multiple oral domains rather than through infection outcomes alone.

Within a structured postoperative pathway, the oral-care formulation is one component of a broader bedside process that also includes timing, frequency, mechanical cleansing, secretion removal, nursing supervision, caregiver participation, and repeated assessment. At the study institution, clindamycin- and chlorhexidine-based formulations were both commonly used after oral cancer surgery according to routine treating-physician practice. Clindamycin and chlorhexidine represent biologically distinct local antimicrobial approaches; topical clindamycin can reduce salivary bacterial counts, whereas chlorhexidine is widely studied for oral antisepsis, plaque control, and gingival health (21–25). Other postoperative formulations, including sodium bicarbonate saline after free-flap surgery, have also been evaluated (7). These practice variations provided an opportunity to examine whether the observed recovery pattern was consistent across commonly used formulations with formulation comparison treated as a secondary exploratory analysis in the present manuscript.

The present prospective observational cohort study examined implementation of a structured early postoperative oral supportive-care pathway after oral cancer resection and free-flap reconstruction and characterized early oral-health recovery during the first two postoperative weeks. The primary focus of the present manuscript was pathway implementation, short-term tolerability, and within-participant change in total and domain-specific OASPOCa scores from POD3 to POD13. Differences between the two routinely used oral-care formulations were examined secondarily and exploratorily; the analyses presented here were not intended to establish formulation superiority, equivalence, or causal pathway efficacy.

## Materials and methods

### Study design and setting

This prospective, single-center observational cohort study was conducted in the otorhinolaryngology ward and intensive-care setting of a regional teaching hospital in central Taiwan. Recruitment was conducted from 1 August 2022 to 31 December 2023. The study prospectively observed delivery of a structured early postoperative oral supportive-care pathway and early oral-health recovery. Data were originally collected prospectively as part of Tsai-Mei Huang’s master’s thesis (26) and were reanalyzed from the participant-level source dataset for the present manuscript. Neither the clinical oral-care formulation nor systemic perioperative management was assigned or modified by the research team.

### Ethics and informed consent

The study was approved by the Institutional Review Board of Tungs’ Taichung MetroHarbor Hospital (IRB no. 111022; approval date, 14 July 2022). Before enrollment, the researcher explained the study purpose and procedures to eligible patients, and all 36 enrolled participants provided written informed consent. Study data were coded and handled confidentially.

### Participants

Eligible participants were adults aged 20 years or older with oral cancer who were admitted for oral cancer resection with free-flap reconstruction, were conscious and able to communicate in Mandarin Chinese or Taiwanese, and provided written informed consent. Exclusion criteria in the original protocol included impaired consciousness or inability to cooperate, a current or recently diagnosed psychiatric disorder, abnormal liver function, and reconstruction with a pedicled or local rotational flap. The psychiatric exclusion was retained as specified in the original protocol. Its stated rationale was to support reliable consent, communication, and participation in repeated care and assessment; however, a diagnosis-based exclusion is broader than an assessment of these abilities and limits the applicability of the findings to patients with psychiatric disorders. “Abnormal liver function” was included as a precautionary criterion because clindamycin (Cleocin), one of the routinely used oral-care formulations, has recognized potential for hepatic adverse effects. The original protocol did not prespecify a numerical biochemical threshold, and no retrospective cutoff was introduced for the present manuscript.

### Routine oral-care formulation use

Both clindamycin-based and chlorhexidine-based formulations were used in routine postoperative care at the study institution. The treating physician selected the formulation according to usual clinical practice and prescribing habits, independently of research sampling. The research team neither assigned the formulation nor altered the physician’s prescription. No standardized clinical criteria were used to select between the two formulations; selection reflected the treating physician’s usual prescribing practice.

For study sampling, a coin toss was used solely to determine which formulation-defined category would contribute the next participant. The next potentially eligible patient already receiving the selected formulation as part of routine care was then considered for formal study eligibility screening; otherwise-eligible patients receiving the other formulation could be bypassed at that sampling step. Eligible patients subsequently provided written informed consent and were enrolled for prospective follow-up, including POD3 and POD13 OASPOCa assessments. Each formulation-defined cohort had a prespecified target of 18 participants. Once one cohort reached 18 participants, further enrollment into that cohort ceased, while accrual to the other cohort continued until its target was reached. Thus, the coin toss determined the observational sampling sequence rather than treatment allocation.

### Shared structured oral supportive-care pathway

Oral supportive care was initiated on the day of surgery as soon as postoperative bedside care commenced, regardless of whether the participant returned to the ward or was transferred to the intensive care unit. Thereafter, care continued throughout the early postoperative period and was performed six times daily in relation to nasogastric-tube feeding. At each session, 30 mL of the routinely used oral-care formulation was applied using a 20-mL syringe positioned approximately 2.5–5 cm (1–2 inches) from the intraoral wound for irrigation, followed by suction drainage and cleansing of the oral cavity with sponge swabs. Formal OASPOCa assessments were performed on POD3 and POD13. The shared pathway is illustrated in **Figure 1**.

**Figure 1:**
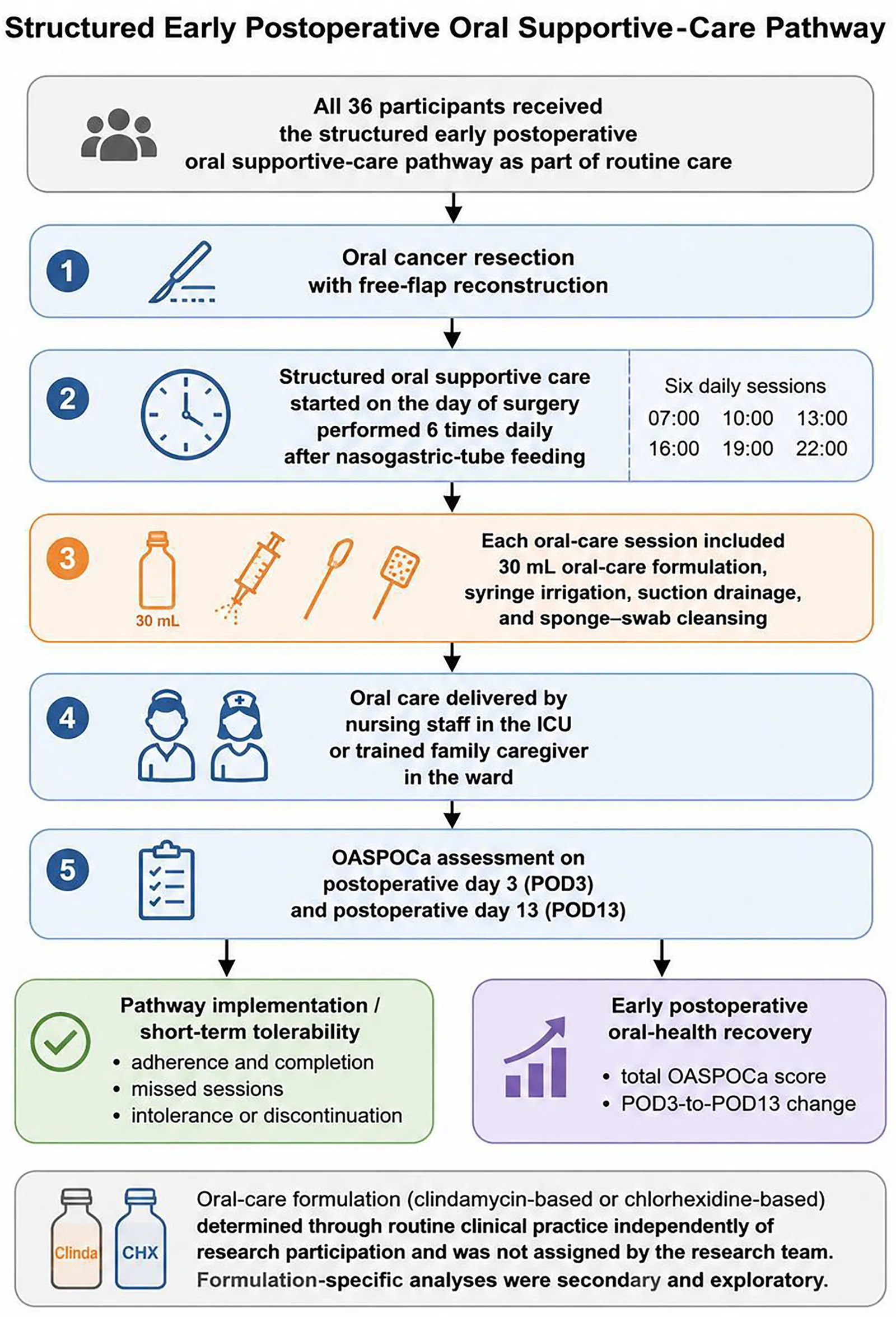
Structured early postoperative oral supportive-care pathway. All 36 participants followed the same structured pathway beginning on the day of surgery. Oral care was performed six times daily after nasogastric-tube feeding and included 30 mL of the routinely used oral-care formulation, syringe irrigation, suction drainage, and sponge-swab cleansing. Nursing staff provided care in the intensive care unit, while trained family caregivers provided ward-based care; ward nurses provided care when caregivers were unavailable. OASPOCa assessments were performed on postoperative days 3 and 13. Feasibility and tolerability were evaluated through adherence, completion, missed sessions, clinically apparent intolerance, and discontinuation, while early oral health recovery was evaluated using total and domain-specific OASPOCa changes. Clindamycin- and chlorhexidine-based formulations reflected routine clinical practice and were examined only in secondary exploratory analyses.

During the first three postoperative days, the same head nurse instructed the primary family caregiver in the oral-cleaning procedure and assessed the caregiver’s technique before independent continuation of care. In the intensive care unit, oral care was performed by unit nurses; on the ward, trained family caregivers performed care, with ward nurses providing care when a family caregiver was unavailable. Caregiver instruction and technique assessment were documented, but caregiver training duration, staffing time, and a validated caregiver competency score were not collected as quantitative outcomes. The research protocol did not override temporary clinical modifications required by postoperative status or feeding management, and exact clock-time deviations were not collected as a separate research variable. Study-specific positioning and suction-pressure parameters were not separately recorded. Systemic antibiotic management followed routine perioperative clinical care and was not assigned or modified by the study. Pathway adherence was prospectively summarized in the study adherence record.

### Oral-care formulations used in routine practice

Clindamycin-based formulation: three 150-mg clindamycin capsules were added to 500 mL of normal saline, yielding a clindamycin concentration of 0.9 mg/mL; 30 mL of the prepared solution was used for each oral-care session. The preparation was replaced daily for use within 24 hours, kept at room temperature during the day of use, and shaken before administration. This formulation reflected pre-existing physician-ordered clinical practice and was not a research-specific investigational product. No study-specific pharmacy compounding procedure or pharmaceutical stability experiment was performed.

Chlorhexidine-based formulation: the commercial product (Parmason Gargle Solution; Panion & BF Biotech Inc., Taiwan) contained chlorhexidine gluconate 0.2% (2 mg/mL) and 4.1% v/v alcohol. Fifteen milliliters of the commercial product were mixed with 15 mL of normal saline, yielding a final chlorhexidine concentration of 0.1% (1 mg/mL) and an approximate final alcohol concentration of 2.05% v/v in the 30-mL administered mixture.

Clinically apparent intolerance or suspected adverse reactions were monitored as part of routine clinical care rather than through a dedicated graded safety framework. The original protocol instructed participants to report symptoms such as oral peeling or irritation, suspected allergic symptoms, gastrointestinal symptoms, jaundice, or oral/throat discomfort to the study physician or nurse. Clinically significant intolerance or a suspected adverse reaction would have prompted discontinuation of the formulation and medical evaluation as appropriate. Because the study required participants to be conscious, communicative, and able to cooperate, individuals unable to provide reliable symptom reporting were not enrolled.

### Outcome assessment

Postoperative oral status was assessed using the Oral Assessment Scale for Post-Operational Patients with Oral Cancer (OASPOCa), developed by Lee et al. (27). The scale contains 10 domains—lips, tongue, gingiva, buccal mucosa, hard palate, floor of the mouth, swallowing, saliva, mouth opening, and surgical-wound condition—with each domain scored from 1 (most favorable) to 3 (most impaired). Total scores range from 10 to 30, with lower scores indicating better oral status. Detailed domain scoring criteria are provided in **Supplementary Table S1**. In patients receiving nasogastric feeding, swallowing was assessed according to the ability to manage and swallow saliva/oral secretions rather than requiring oral food intake. Mouth opening was classified using the instrument’s millimeter categories (≥31 mm, 16–30 mm, and ≤15 mm), with finger-breadth/tongue-depressor equivalents used operationally. The reconstructed flap and suture line were evaluated within the surgical-wound domain using postoperative criteria including flap appearance, warmth, capillary refill, wound approximation, and the presence of blood, clot, secretions, or other concerning findings.

Published psychometric properties of OASPOCa include a content validity index of 1.00, inter-rater intraclass correlation coefficients of 0.97 for the lips and tongue and 1.00 for the remaining eight domains, and a Cronbach’s alpha of 0.72; oral-status scores were also reported to correlate inversely with oral comfort (27). These published reliability estimates should not be interpreted as study-specific calibration. In the present study, all formal OASPOCa assessments were performed by the same head nurse, who had clinical experience in postoperative oral cancer care but did not undergo a separate study-specific standardized OASPOCa calibration exercise. Blinding to the oral-care formulation was not feasible. Assessments were conducted during the day shift, one hour after oral care. POD3 represented an early postoperative assessment after pathway initiation, whereas POD13 represented the later assessment at the end of the structured early postoperative observation period.

### Study outcomes

The POD3 and POD13 OASPOCa assessments and the comparison of the two oral-care formulations were specified before recruitment in the original prospective study plan. The original study also used an *a priori* G*Power calculation to set a target total sample of 36 participants, with 18 planned in each formulation-defined cohort; no retrospective power calculation was performed for the present manuscript. For the current manuscript, the prospectively recorded adherence, assessment-completion, and clinically apparent intolerance information was organized as implementation and short-term tolerability outcomes. Longitudinal within-participant change in total and domain-specific OASPOCa scores from POD3 to POD13 was used to characterize early oral-health recovery. The prespecified formulation comparison was retained but interpreted conservatively as secondary and exploratory because formulation use reflected routine clinical practice rather than research-directed treatment assignment.

### Other variables

Prospectively recorded participant characteristics included sex, age, education, smoking history, alcohol-use history, betel-quid use, and initial versus recurrent oral cancer. Hospital length of stay was recorded categorically and is presented separately as postoperative clinical context. Detailed tumor subsite, TNM stage, resection extent, flap subtype, prior radiotherapy/chemoradiotherapy, tracheostomy status, comorbidity burden, nutritional status, and baseline dentition were not collected as standardized study variables and were not retrospectively reconstructed for the present analysis.

### Statistical analysis

Categorical variables were summarized as frequencies and percentages and continuous variables as mean (standard deviation [SD]) or median (interquartile range [IQR]), as appropriate. Participant characteristics were presented descriptively without formal baseline-equivalence testing. For the overall cohort, total OASPOCa scores were calculated by summing the 10 domain scores at POD3 and POD13. Individual improvement was defined as POD3 total score minus POD13 total score, so positive values indicated improvement. Cohort-level change was summarized with a paired t test and a 95% confidence interval, with median change and IQR additionally reported.

For the secondary formulation-specific analysis, the between-cohort difference in mean individual improvement was estimated using Welch’s two-sample t test with a 95% confidence interval. A bias-corrected and accelerated (BCa) bootstrap confidence interval was calculated using 100,000 independent within-cohort resamples with replacement and a fixed random seed of 20260811. A two-sided exact permutation test was calculated without Monte Carlo sampling: because the 36 observed integer improvement scores were divided into two groups of 18, a dynamic-programming algorithm counted the exact permutation distribution across all 9,075,135,300 possible 18-person label allocations by subset sum. For domain-level analyses, each participant was classified as improved, unchanged, or worsened; Fisher’s exact test compared improved versus not improved between formulation-defined cohorts, with absolute differences in improvement proportions and 95% confidence intervals calculated using the Newcombe score method without continuity correction and reported descriptively. No adjustment for multiple comparisons was applied, and domain-level P values were interpreted as exploratory. Resampling assessed statistical robustness only and did not remove confounding inherent to the observational formulation comparison. Original data management was performed using IBM SPSS Statistics for Windows, version 22.0 (IBM Corp., Armonk, NY, USA); revised analyses were reproduced from the participant-level source dataset using Python 3.12, NumPy 2.3.5, SciPy 1.17.0, and openpyxl 3.1.5. Reproducible verification code is provided as **Supplementary Code S1**.

## Results

### Participant flow

After application of the prospective formulation-category sampling procedure, 38 patients entered formal study eligibility screening and 36 were enrolled with written informed consent. Two patients were excluded before enrollment: one because of abnormal liver function and one because the planned surgical procedure was changed such that the patient no longer met the prespecified operative eligibility criteria. The 38 formally screened participants do not represent all oral-cancer surgery patients treated at the institution during the 17-month recruitment period because otherwise-eligible patients receiving the non-selected routine formulation could be bypassed at a given sampling step. All 36 enrolled participants entered the structured early postoperative oral supportive-care pathway, completed POD3 and POD13 OASPOCa assessments, and were included in the final analysis (**Figure 2**).

**Figure 2:**
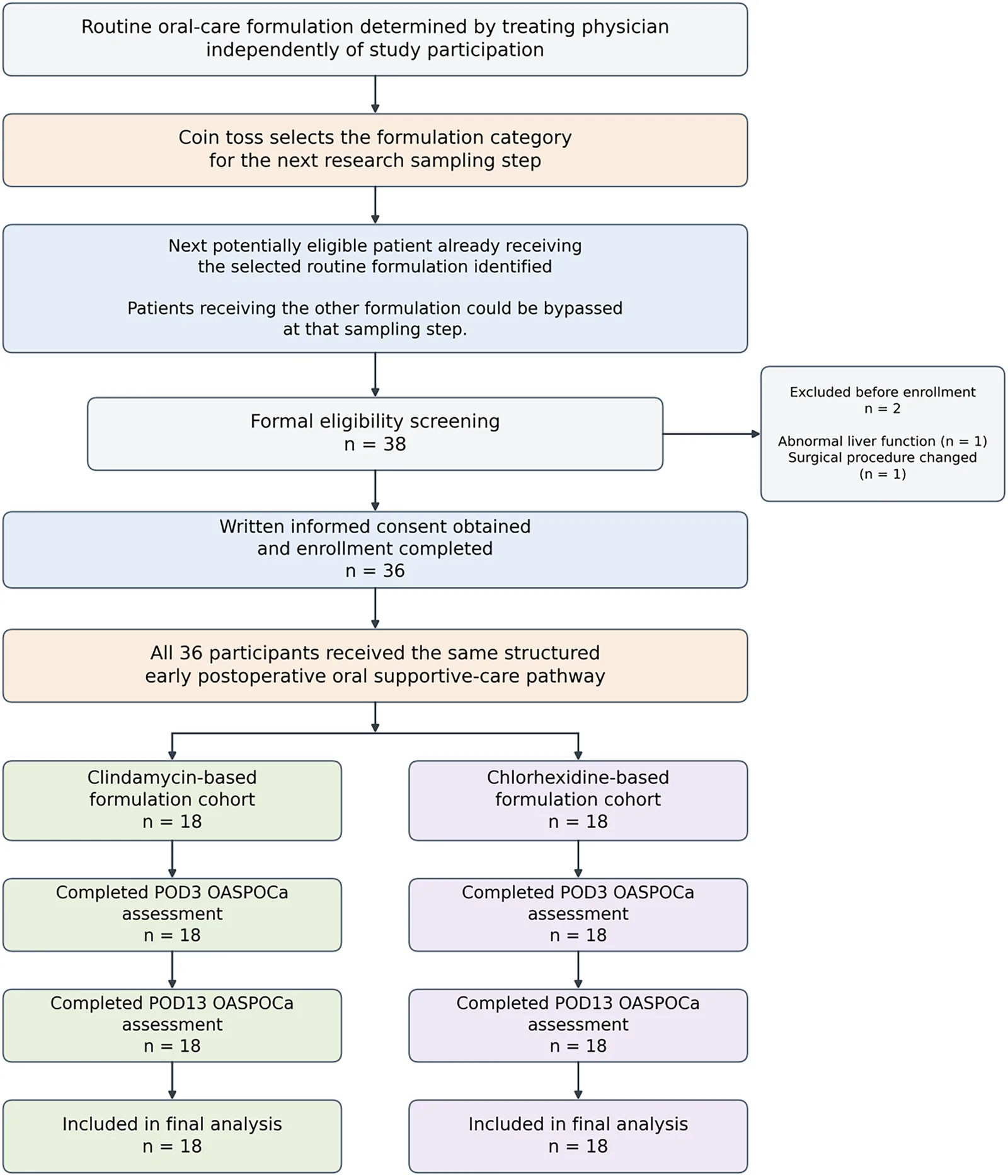
Participant flow and observational sampling framework of the prospective cohort. Routine oral-care formulation was determined by treating-physician practice independently of study participation. The coin toss selected only the formulation-defined category for the next research sampling step; the next potentially eligible patient already receiving that routine formulation was then considered for formal study screening. Otherwise-eligible patients receiving the other formulation could be bypassed at that sampling step. After 38 patients entered formal screening, one was excluded because of abnormal liver function and one because a change in the planned surgical procedure meant that the patient no longer met the prespecified operative eligibility criteria; 36 participants subsequently provided written informed consent. Each formulation-defined cohort had a prespecified target of 18 participants. All 36 participants completed POD3 and POD13 OASPOCa assessments and were included in the final analysis. Formulation-specific analyses were secondary and exploratory.

### Participant characteristics

Participant characteristics according to the routinely used oral-care formulation are summarized descriptively in **Table 1**. Of the 36 participants, 35 (97.2%) were men. Eighteen (50.0%) were aged 40–59 years, 13 (36.1%) were aged 60–69 years, four (11.1%) were aged at least 70 years, and one (2.8%) was aged 20–39 years. Twenty-one participants (58.3%) had an initial oral cancer diagnosis and 15 (41.7%) had recurrent disease. No formal hypothesis testing of baseline characteristics was performed.

**Table 1:** Prospectively recorded participant characteristics by routinely used oral-care formulation (N = 36).

Characteristic	Clindamycin-based formulation (n = 18)	Chlorhexidine-based formulation (n = 18)	Total (n = 36)
Sex			
Male	18 (100.0)	17 (94.4)	35 (97.2)
Female	0 (0.0)	1 (5.6)	1 (2.8)
Age			
20–39 years	0 (0.0)	1 (5.6)	1 (2.8)
40–59 years	11 (61.1)	7 (38.9)	18 (50.0)
60–69 years	5 (27.8)	8 (44.4)	13 (36.1)
≥70 years	2 (11.1)	2 (11.1)	4 (11.1)
Education			
Primary school or less	1 (5.6)	3 (16.7)	4 (11.1)
Junior high school	9 (50.0)	2 (11.1)	11 (30.6)
Senior high/vocational	7 (38.9)	10 (55.6)	17 (47.2)
College or higher	1 (5.6)	3 (16.7)	4 (11.1)
Smoking			
Never	1 (5.6)	2 (11.1)	3 (8.3)
Former/current	17 (94.4)	16 (88.9)	33 (91.7)
Alcohol			
Never	1 (5.6)	3 (16.7)	4 (11.1)
Former/current	17 (94.4)	15 (83.3)	32 (88.9)
Betel quid			
Never	8 (44.4)	9 (50.0)	17 (47.2)
Former/current	10 (55.6)	9 (50.0)	19 (52.8)
Diagnosis			
Initial	10 (55.6)	11 (61.1)	21 (58.3)
Recurrent	8 (44.4)	7 (38.9)	15 (41.7)

Values are n (%). Oral-care formulation groups reflect routine physician practice; the formulation was not assigned by the research team. Characteristics are presented descriptively, and no formal baseline-equivalence testing was performed.

### Postoperative clinical context

Hospital length of stay was retained in the source dataset as categories rather than exact individual durations. In the clindamycin-defined cohort, 2/18 participants stayed 10–15 days, 6/18 stayed 16–20 days, and 10/18 stayed ≥21 days; corresponding counts in the chlorhexidine-defined cohort were 11/18, 5/18, and 2/18. This difference is presented as postoperative clinical context rather than a baseline characteristic or a formulation effect and may reflect unmeasured differences in case complexity, postoperative course, discharge readiness, or clinical practice. All participants remained hospitalized and available for inpatient POD13 assessment.

### Protocol delivery, adherence, and tolerability

All 36 participants completed the paired POD3 and POD13 OASPOCa assessments. The adherence sheet recorded 32 of 36 participants (88.9%) as completing all scheduled daily oral-care sessions; four participants had one missed-session record, with two in each formulation-defined cohort. The archived adherence dataset did not retain the specific reasons for these four isolated missed sessions, did not separately classify medically withheld sessions, and did not preserve whether each individual session was verified by direct nursing observation or caregiver report. We therefore do not retrospectively assign reasons or a more granular session denominator that was not recorded. No participant was recorded as using an additional oral-cleaning product. No clinically significant formulation-related intolerance or discontinuation was recorded; adverse-event surveillance was clinical rather than based on a dedicated graded safety log. These implementation and short-term tolerability findings are summarized in **Table 2**.

**Table 2:** Pathway implementation, short-term tolerability, and early oral-health recovery outcomes in the overall cohort (N = 36).

Outcome	Overall cohort result	Analysis/definition
Completed POD3 and POD13 OASPOCa assessments	36/36 (100%)	Feasibility: paired assessment completion
Recorded as completing all scheduled daily oral-care sessions	32/36 (88.9%)	Complete participant-level adherence according to the archived adherence record
Missed one scheduled oral-care session	4/36 (11.1%)	Four isolated missed-session records; specific reasons were not retained in the archived adherence dataset
Use of additional oral-cleaning products	0/36 (0%)	Recorded during the study period
Clinically significant formulation-related intolerance recorded	0/36 (0%)	Clinical monitoring; no dedicated graded adverse-event log
Formulation-related discontinuation because of intolerance/adverse reaction	0/36 (0%)	No participant discontinued because of clinically significant intolerance
POD3 total OASPOCa, mean (SD)	14.97 (2.79)	Lower score indicates better oral status
POD13 total OASPOCa, mean (SD)	11.44 (1.65)	Lower score indicates better oral status
Within-participant improvement, mean (SD)	3.53 (3.15)	POD3 minus POD13
Mean improvement, 95% CI; paired P value	2.46 to 4.59; P < 0.001	Two-sided paired t test; temporal change, not causal pathway effect
Within-participant improvement, median (IQR)	3.0 (1.75–6.0)	POD3 minus POD13
Remained hospitalized and available for inpatient POD13 assessment	36/36 (100%)	POD13 assessment completed during hospitalization

OASPOCa, oral assessment scale for post-operational patients with oral cancer; CI, confidence interval; IQR, interquartile range; SD, standard deviation. Assessment completion and oral-care adherence are reported separately. The archived adherence record did not retain the specific reasons for the four isolated missed sessions. Clinically apparent intolerance was monitored in routine care but was not collected using a dedicated graded safety framework. Oral care began before the POD3 assessment; therefore, paired change describes temporal recovery and does not estimate causal pathway efficacy.

### Early oral-health recovery

Across all 36 participants, mean total OASPOCa score decreased from 14.97 (SD 2.79) on postoperative day 3 to 11.44 (SD 1.65) on postoperative day 13. Mean within-participant improvement was 3.53 points (SD 3.15; 95% CI, 2.46–4.59; paired P < 0.001), with a median improvement of 3.0 points (IQR 1.75–6.0) (**Table 2**). These findings characterize early temporal improvement in oral status during the structured supportive-care period. Formulation-specific total-score results are presented in **Table 3**.

**Table 3:** Exploratory comparison of change in total OASPOCa score by routinely used oral-care formulation.

Outcome	Clindamycin-based formulation (n = 18)	Chlorhexidine-based formulation (n = 18)	Between-formulation difference (95% CI)	P value
POD3 total OASPOCa, mean (SD)	15.17 (3.07)	14.78 (2.56)	—	—
POD13 total OASPOCa, mean (SD)	11.44 (2.12)	11.44 (1.04)	—	—
Improvement, mean (SD)†	3.72 (3.54)	3.33 (2.79)	0.39 (−1.78, 2.55)	0.717
Improvement, median (IQR)	4.0 (1.5–5.75)	2.0 (2.0–6.0)	—	—

† Improvement = postoperative day-3 total OASPOCa score minus postoperative day-13 total OASPOCa score; positive values indicate improvement. Lower OASPOCa scores indicate better oral status. The between-formulation difference in mean improvement was estimated using Welch’s two-sample t test. Formulation use reflected routine treating-physician practice, and these comparisons were secondary and exploratory. OASPOCa, oral assessment scale for post-operational patients with oral cancer; CI, confidence interval; IQR, interquartile range; SD, standard deviation.

### Domain-level oral-health recovery

Across the full cohort, at least some participants improved from POD3 to POD13 in each of the 10 OASPOCa domains, while other participants remained unchanged or worsened (**Table 4**). The proportion improving ranged from 22.2% for swallowing to 55.6% for saliva; worsening was uncommon. Full score-1/2/3 distributions at both assessments are provided in **Supplementary Table S2**. Importantly, opportunity for improvement differed at POD3. For saliva, only 3/18 participants in the clindamycin-defined cohort were already in the most favorable category at POD3 compared with 10/18 in the chlorhexidine-defined cohort, limiting direct interpretation of subsequent improvement proportions.

**Table 4:** Domain-level changes in OASPOCa scores in the overall cohort and by routinely used oral-care formulation.

OASPOCa domain	Overall cohort (n = 36) Improved/Unchanged/Worsened	Clindamycin-based formulation (n = 18) Improved/Unchanged/Worsened	Chlorhexidine-based formulation (n = 18) Improved/Unchanged/Worsened	Difference in improvement, % (95% CI)	Fisher P
Lips	9/27/0	5/13/0	4/14/0	+5.6 (−22.1 to 32.2)	1.000
Tongue	17/16/3	9/7/2	8/9/1	+5.6 (−24.7 to 34.5)	1.000
Gingiva	14/21/1	9/8/1	5/13/0	+22.2 (−9.0 to 48.2)	0.305
Buccal mucosa	13/20/3	6/10/2	7/10/1	−5.6 (−33.8 to 23.9)	1.000
Hard palate	14/19/3	5/11/2	9/8/1	−22.2 (−48.2 to 9.0)	0.305
Floor of mouth	13/22/1	7/10/1	6/12/0	+5.6 (−23.9 to 33.8)	1.000
Swallowing	8/27/1	4/13/1	4/14/0	0.0 (−26.5 to 26.5)	1.000
Saliva	20/16/0	13/5/0	7/11/0	+33.3 (+1.1 to +57.4)	0.092
Mouth opening	14/18/4	9/8/1	5/10/3	+22.2 (−9.0 to 48.2)	0.305
Surgical wound	18/16/2	10/7/1	8/9/1	+11.1 (−19.8 to 39.2)	0.740

Improvement was defined as a lower OASPOCa domain score on postoperative day 13 than on postoperative day 3; unchanged as the same score; and worsened as a higher score. Overall-cohort counts were obtained by combining the two routine formulation groups. Difference in improvement represents the absolute percentage-point difference in the proportion improved (clindamycin minus chlorhexidine). Fisher’s exact test compares improved versus not improved for the secondary exploratory formulation analysis. Domain-level formulation analyses are exploratory, and P values should be interpreted descriptively. OASPOCa, oral assessment scale for post-operational patients with oral cancer; CI, confidence interval. Confidence intervals for differences in improvement proportions were calculated using the Newcombe score method without continuity correction.

### Exploratory formulation-specific analysis

Eighteen participants received the clindamycin-based formulation and 18 received the chlorhexidine-based formulation as part of routine clinical care. Mean improvement in total OASPOCa score was 3.72 points (SD 3.54) and 3.33 points (SD 2.79), respectively. The exploratory between-formulation difference was 0.39 points (95% CI, −1.78 to 2.55; Welch P = 0.717) (**Table 3**). After reproducible reanalysis with a fixed random seed, the BCa bootstrap 95% confidence interval was −1.83 to 2.22; the exact two-sided permutation P value was 0.757. Domain-level exploratory comparisons are shown in **Table 4**. The largest numerical difference occurred for saliva, but this finding is interpreted only as hypothesis-generating because of the small sample, multiple exploratory domains, and different baseline opportunity for improvement.

## Discussion

### Principal findings

This prospective observational cohort study characterized delivery of a structured early postoperative oral supportive-care pathway and early oral-health recovery after oral cancer resection and free-flap reconstruction. All 36 participants completed paired POD3/POD13 assessments, 88.9% were recorded as completing all scheduled daily sessions in the archived adherence record, and no clinically significant formulation-related intolerance or discontinuation was identified. Total OASPOCa scores improved over time, and at least some participants improved in every domain. The routinely used clindamycin- and chlorhexidine-based formulations were compared only secondarily and exploratorily, with no clear overall formulation-specific advantage.

A central contribution is the detailed description of care delivery during a period when patients often have limited capacity for independent oral hygiene. The pathway began on the day of surgery and combined repeated irrigation and cleansing, secretion removal, caregiver instruction, nursing supervision, and serial multidimensional assessment. The high recorded participant-level adherence and complete paired assessments suggest that this bedside process was practicable in the study setting. However, session-level verification was not retained, and the absence of recorded clinically significant intolerance provides only limited short-term tolerability information. These observations do not establish pathway efficacy or safety.

### Clinical relevance of early postoperative oral supportive care

Existing evidence supports the clinical relevance of perioperative oral care in patients undergoing major cancer surgery. A 2024 systematic review and meta-analysis reported associations between professional perioperative oral hygiene care and a lower risk of surgical-site infection and shorter hospital stay (5). In head and neck free-flap reconstruction, professional preoperative oral care has also been associated with lower surgical-site infection risk (2), while inadequate plaque control has been linked to postoperative pneumonia (6). More recent head and neck cancer data further support continuity of perioperative oral care across the surgical episode, with postoperative oral care associated with a lower risk of surgical-site infection (28). Postoperative infections may also have important downstream consequences, including plate exposure, reoperation, prolonged hospitalization, and delayed adjuvant radiotherapy (8, 9). Much of this literature, however, has focused on infection prevention or preoperative dental intervention. The present study extends the clinical perspective to the immediate postoperative bedside period, when the patient’s ability to perform oral hygiene is particularly limited and care must be integrated into feeding, wound management, secretion control, and functional recovery.

Serial OASPOCa assessment captured recovery across mucosal, salivary, swallowing, mouth-opening, and surgical-wound domains (27). This multidimensional approach is useful because improvement is not uniform across postoperative functions. However, no established minimally clinically important difference is available for OASPOCa, so the observed score change should be interpreted by its magnitude and uncertainty rather than against an unvalidated response threshold. In addition, the POD13 mean of 11.44 approached the theoretical minimum of 10, indicating a potential floor effect that may limit measurable improvement at the later assessment.

### Exploratory interpretation of oral-care formulation findings

The two oral-care formulations reflected routine physician practice rather than research-directed assignment, so their comparison remains secondary and exploratory. The between-formulation difference in total-score improvement was small and imprecisely estimated, and the resampling analyses assess statistical robustness rather than control of confounding. The numerical saliva difference is particularly susceptible to differing baseline opportunity for improvement and multiple-domain testing. Possible effects of oral dryness, local antimicrobial exposure, or mucosal irritation are biologically plausible but were not measured and should be regarded as speculative.

Topical clindamycin and chlorhexidine have distinct antimicrobial properties, but the present study did not measure microbial burden, antimicrobial resistance, local drug concentration, salivary physiology, or microbiome composition (21–25). Repeated topical clindamycin exposure reflected pre-existing clinical practice rather than a study-directed recommendation. Accordingly, these data should not be interpreted as evidence supporting routine topical antibiotic use; antimicrobial stewardship and formulation-specific benefit require dedicated controlled evaluation.

### Clinical implications

The most directly applicable finding is the feasibility of organizing early postoperative oral care as a defined bedside process with specified timing, mechanical cleansing, caregiver instruction, nursing supervision, and serial assessment. Such standardization may reduce variability in who delivers care and how recovery is documented. The current data do not identify a preferred formulation; rather, the pathway provides a practical framework within which future oral-care agents or supportive-care strategies can be evaluated under more controlled conditions.

Future studies should incorporate a true pretreatment baseline, controlled or randomized formulation assignment, prespecified safety monitoring, and clinically relevant outcomes such as infection, wound complications, function, microbiology, and patient-reported recovery.

### Strengths and limitations

Strengths include prospective observation of a complete 36-participant cohort, initiation of care on the day of surgery, integration of caregiver and nursing roles, complete paired OASPOCa data, and a prospectively maintained adherence record. The study therefore provides a reproducible description of a structured early oral-supportive-care process across ward and intensive-care settings.

Several limitations define the scope of inference. This was a single-center, predominantly male cohort. Formulation use reflected routine physician practice rather than random assignment, so formulation-specific estimates remain vulnerable to measured and unmeasured confounding. Detailed tumor subsite, TNM stage, resection extent, flap subtype, prior radiotherapy/chemoradiotherapy, tracheostomy status, comorbidity burden, nutritional status, and baseline dentition were not collected as standardized research variables and were not reconstructed retrospectively. The marked difference in categorical hospital length of stay between formulation-defined cohorts may reflect additional unmeasured clinical heterogeneity. All OASPOCa assessments were performed by one experienced but unblinded head nurse without a separate study-specific calibration exercise. POD3 was not a pretreatment baseline, no untreated or usual-care control group was included, and follow-up ended at POD13. Adverse-event monitoring was clinical rather than based on a dedicated graded safety framework, and the archived adherence record did not retain reasons for the four isolated missed sessions. Domain analyses were exploratory with sparse cells and multiple comparisons. These limitations preclude causal inference and require confirmation in larger controlled studies.

## Conclusion

In this prospective observational cohort, a structured early postoperative oral supportive-care pathway incorporating repeated cleansing, nursing supervision, caregiver participation, and serial multidimensional assessment was implemented with high recorded participant-level adherence after oral cancer resection and free-flap reconstruction. No clinically significant formulation-related intolerance or discontinuation was recorded, providing preliminary short-term tolerability information rather than definitive safety evidence. Oral status improved from POD3 to POD13, but this temporal recovery cannot be attributed solely to the pathway because oral care had begun before the first formal assessment and no untreated control group was included. Secondary exploratory analyses showed no clear overall advantage of either routinely used oral-care formulation. These findings support the pathway as a practical framework for future controlled evaluation rather than as evidence of causal efficacy or formulation superiority.

## Data Availability

The datasets presented in this article are not readily available because De-identified participant-level data supporting the conclusions of this article may be made available by the authors upon reasonable request, subject to approval by the Institutional Review Board of Tungs’ Taichung MetroHarbor Hospital and applicable ethical and privacy requirements. Requests to access the datasets should be directed to tsaistella111@nchu.edu.tw.
