# Supplementary material for: Implementation of a structured early oral supportive-care pathway and early oral health recovery after oral cancer resection and free-flap reconstruction: a prospective observational cohort study
Source: Front Oncol. 2026 Oct 20;16:1981326. doi: 10.3389/fonc.2026.1981326 (PMC13727435; doi:10.3389/fonc.2026.1981326)
Supplement: Supplementary file 1 [file Table1.docx]

Supplementary Material

**Supplementary Table S1. OASPOCa domain scoring criteria used in the study**

| **Domain** | **Score 1 (most favorable)** | **Score 2** | **Score 3 (most impaired)** |
| --- | --- | --- | --- |
| Lips | Pink, moist, soft | Blood staining or peeling | Cracking, spontaneous bleeding, or erythematous/swollen appearance |
| Tongue | Pink, moist, soft | Thin coating and/or viscous sputum/saliva | Dry/cracked tongue, thick coating, or erythematous/swollen appearance |
| Gingiva | Pink | Blood staining, mucus/sputum clot, or viscous saliva | Erythema/swelling, ulceration, or bleeding |
| Buccal mucosa | Pink | Blood staining, viscous sputum/sputum clot, or viscous saliva | Dryness, ulceration, bleeding, or erythema/swelling |
| Hard palate | Pink | Blood staining, viscous sputum/sputum clot, or viscous saliva | Dryness, ulceration, bleeding, or erythema/swelling |
| Floor of mouth | Pink | Blood staining, viscous sputum/sputum clot, or viscous saliva | Dryness, ulceration, bleeding, or erythema/swelling |
| Swallowing | Able to swallow saliva/oral contents smoothly | Saliva/oral contents catch in the throat or are difficult to swallow | Unable to swallow and/or choking or drooling |
| Saliva | Watery and clear | Viscous or foamy | No saliva / marked oral dryness |
| Mouth opening | ≥31 mm | 16–30 mm | ≤15 mm |
| Surgical wound | Flap full and warm; pink; capillary refill 1–2 s; no blood staining or clot at wound | Blood clot or secretion at flap suture line and/or suboptimal wound approximation; blood/clot accumulation | Concerning flap or wound findings such as pallor/dryness/coolness with delayed refill, congestion/cyanotic discoloration with rapid refill, bleeding, purulent secretion, or malodor |

Scoring criteria are reproduced in concise English from the OASPOCa instrument used in the original study. For mouth opening, finger-breadth/tongue-depressor equivalents were used operationally in the original instrument. In nasogastric-fed participants, the swallowing domain was evaluated according to saliva/oral-secretion handling rather than requiring oral food intake.

**Supplementary Table S2. OASPOCa score distributions on POD3 and POD13 by routine formulation**

| **Domain** | **Clindamycin POD3 score 1 / 2 / 3** | **Clindamycin POD13 score 1 / 2 / 3** | **Chlorhexidine POD3 score 1 / 2 / 3** | **Chlorhexidine POD13 score 1 / 2 / 3** | **Overall POD3 score 1 / 2 / 3** | **Overall POD13 score 1 / 2 / 3** |
| --- | --- | --- | --- | --- | --- | --- |
| Lips | 13 (72.2%) / 5 (27.8%) / 0 (0.0%) | 18 (100.0%) / 0 (0.0%) / 0 (0.0%) | 12 (66.7%) / 6 (33.3%) / 0 (0.0%) | 16 (88.9%) / 2 (11.1%) / 0 (0.0%) | 25 (69.4%) / 11 (30.6%) / 0 (0.0%) | 34 (94.4%) / 2 (5.6%) / 0 (0.0%) |
| Tongue | 9 (50.0%) / 9 (50.0%) / 0 (0.0%) | 16 (88.9%) / 2 (11.1%) / 0 (0.0%) | 10 (55.6%) / 8 (44.4%) / 0 (0.0%) | 17 (94.4%) / 1 (5.6%) / 0 (0.0%) | 19 (52.8%) / 17 (47.2%) / 0 (0.0%) | 33 (91.7%) / 3 (8.3%) / 0 (0.0%) |
| Gingiva | 9 (50.0%) / 8 (44.4%) / 1 (5.6%) | 16 (88.9%) / 2 (11.1%) / 0 (0.0%) | 11 (61.1%) / 6 (33.3%) / 1 (5.6%) | 16 (88.9%) / 2 (11.1%) / 0 (0.0%) | 20 (55.6%) / 14 (38.9%) / 2 (5.6%) | 32 (88.9%) / 4 (11.1%) / 0 (0.0%) |
| Buccal mucosa | 12 (66.7%) / 6 (33.3%) / 0 (0.0%) | 16 (88.9%) / 2 (11.1%) / 0 (0.0%) | 11 (61.1%) / 7 (38.9%) / 0 (0.0%) | 17 (94.4%) / 1 (5.6%) / 0 (0.0%) | 23 (63.9%) / 13 (36.1%) / 0 (0.0%) | 33 (91.7%) / 3 (8.3%) / 0 (0.0%) |
| Hard palate | 13 (72.2%) / 5 (27.8%) / 0 (0.0%) | 16 (88.9%) / 2 (11.1%) / 0 (0.0%) | 9 (50.0%) / 9 (50.0%) / 0 (0.0%) | 17 (94.4%) / 1 (5.6%) / 0 (0.0%) | 22 (61.1%) / 14 (38.9%) / 0 (0.0%) | 33 (91.7%) / 3 (8.3%) / 0 (0.0%) |
| Floor of mouth | 11 (61.1%) / 7 (38.9%) / 0 (0.0%) | 17 (94.4%) / 1 (5.6%) / 0 (0.0%) | 10 (55.6%) / 8 (44.4%) / 0 (0.0%) | 16 (88.9%) / 2 (11.1%) / 0 (0.0%) | 21 (58.3%) / 15 (41.7%) / 0 (0.0%) | 33 (91.7%) / 3 (8.3%) / 0 (0.0%) |
| Swallowing | 13 (72.2%) / 5 (27.8%) / 0 (0.0%) | 17 (94.4%) / 0 (0.0%) / 1 (5.6%) | 14 (77.8%) / 3 (16.7%) / 1 (5.6%) | 18 (100.0%) / 0 (0.0%) / 0 (0.0%) | 27 (75.0%) / 8 (22.2%) / 1 (2.8%) | 35 (97.2%) / 0 (0.0%) / 1 (2.8%) |
| Saliva | 3 (16.7%) / 15 (83.3%) / 0 (0.0%) | 16 (88.9%) / 2 (11.1%) / 0 (0.0%) | 10 (55.6%) / 7 (38.9%) / 1 (5.6%) | 17 (94.4%) / 1 (5.6%) / 0 (0.0%) | 13 (36.1%) / 22 (61.1%) / 1 (2.8%) | 33 (91.7%) / 3 (8.3%) / 0 (0.0%) |
| Mouth opening | 2 (11.1%) / 12 (66.7%) / 4 (22.2%) | 8 (44.4%) / 9 (50.0%) / 1 (5.6%) | 5 (27.8%) / 8 (44.4%) / 5 (27.8%) | 5 (27.8%) / 11 (61.1%) / 2 (11.1%) | 7 (19.4%) / 20 (55.6%) / 9 (25.0%) | 13 (36.1%) / 20 (55.6%) / 3 (8.3%) |
| Surgical wound | 7 (38.9%) / 11 (61.1%) / 0 (0.0%) | 16 (88.9%) / 2 (11.1%) / 0 (0.0%) | 10 (55.6%) / 8 (44.4%) / 0 (0.0%) | 17 (94.4%) / 1 (5.6%) / 0 (0.0%) | 17 (47.2%) / 19 (52.8%) / 0 (0.0%) | 33 (91.7%) / 3 (8.3%) / 0 (0.0%) |

Values are n (%), shown as score 1 / score 2 / score 3. Lower scores indicate more favorable oral status. The distributions make the baseline opportunity for improvement visible; for example, at POD3, 3/18 participants in the clindamycin-defined cohort and 10/18 in the chlorhexidine-defined cohort were already in the most favorable saliva category.
